# Celastrol increases anoikis sensitivity to suppress triple-negative breast cancer via EGFR pathway and p-EMT state regulation

**DOI:** 10.3389/fphar.2026.1747871

**Published:** 2026-03-02

**Authors:** Jue Yang, Xiangpeng Wang, Xiuyun Bai, Rongjun Deng, Wanting Ye, Qiqiong Liu, Ying Xing, Yuxing Yao, Aiping Lyu, Cheng Lu, Yuanyan Liu

**Affiliations:** 1 School of Materia Medica, Beijing University of Chinese Medicine, Beijing, China; 2 School of Chinese Medicine, Hong Kong Baptist University, Kowloon, China; 3 Institute of Basic Research in Clinical Medicine, China Academy of Chinese Medical Sciences, Beijing, China

**Keywords:** anoikis, celastrol, circulating tumor cells, triple-negative breast cancer, tumor metastasis

## Abstract

Faced with the highly malignant threat of metastatic triple-negative breast cancer (TNBC), the effect of traditional chemical agents is limited, and new anti-metastasis drug remains to be explored. Celastrol (Cel) is a bioactive compound derived from *Tripterygium wilfordii* with significant anti-neoplastic effects in various cancers. In this study, we investigated the potential anti-metastatic effects of Cel on anoikis resistant TNBC cells (TNBC-AR) cells, including MDA-MB-231-AR cells and BT-549-AR cells. Using CCK-8 and colony formation assay, we demonstrated that Cel could inhibit the proliferation of MDA-MB-231-AR cells and BT-549-AR cells with IC_50_ value of 1.510 μM and 1.673 μM, respectively. The results of wound healing and transwell assays showed that Cel could potently inhibit the invasion and migration of TNBC-AR cells. Aggregation and flow cytometry experiments showed that Cel could inhibit the clusters formation and enhance the anoikis of TNBC-AR cells on the suspension conditions. Then we conducted bioinformatics analysis, Western blotting, and intervention experiments to explore the molecular mechanisms of Cel’s anti-metastasis effects. The results of these experiments discovered that Cel treatment suppressed the p-EMT state in TNBC-AR cells, and this effect correlated with a reduction in EGFR/MEK/ERK pathway activation. Our findings suggest that Cel may be a promising candidate for therapeutic treatments of metastatic TNBC.

## Graphical Abstract

**Figure:**
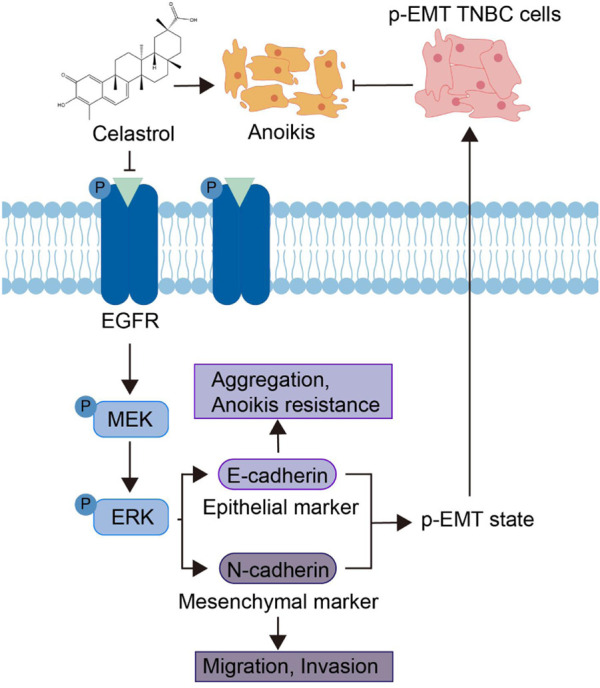

## Highlights

- Celastrol has been proved to be effective in inhibiting the invasion and metastasis of triple-negative breast cancer.
- Targeting EGFR and p-EMT to enhance anoikis provides a new therapeutic strategy for curbing metastasis in triple-negative breast cancer.

## Introduction

Breast cancer remains the most prevalent and fatal cancer among women, which is classified by the expression of hormone receptors (HR) and human epidermal growth factor receptor type 2 (HER2) (Ta et al., 2025; Yusein-Myashkova et al., 2025; Bocachica-Adorno et al., 2025). Triple-negative breast cancer (TNBC), the most aggressive and metastatic subtype of breast cancer, is characterized by the absence of estrogen and progesterone receptors and HER2 (Bocachica-Adorno et al., 2025). Due to the lack of above targets, the treatment of TNBC is still challenging for limited therapies and high risk of metastasis. In clinical, the 5-year survival rate of patients with metastatic TNBC is only 10% (Hsu et al., 2022).

Metastasis is the deadliest event during tumor development that comprises of multiple steps, including invasion into surrounding tissues, circulation through blood system and colonization in distant organs (Majidpoor and Mortezaee, 2021). To achieve this complex process, a variety of molecular mechanisms are profoundly involved in the metastatic cascade, such as epithelial-mesenchymal transition (EMT) and anoikis resistance (Meng et al., 2024). During EMT process, tumor cells change their cell morphology from epithelial to mesenchymal. This process is often accompanied by loss of cell polarity and increased migratory ability, which allow tumor cells to detach from primary tissues and intravasate into circulatory system (Cao et al., 2023). Recently, EMT process is found to be not a binary process. For instance, tumor cells undergoing partial epithelial-mesenchymal transition (p-EMT) has been reported to express both epithelial and mesenchymal markers. Tumor cells with p-EMT state can be better adapted to stress situations, such as anoikis (Fonseca et al., 2023). Anoikis is a type of programmed cell death that emerges once cells detach from initial extracellular matrix (ECM) to maintain tissue homeostasis. However, tumor cells with anoikis resistance can overcome anoikis and survive in the circulatory system to migrate to distant tissues or organs (Dong et al., 2024). Therefore, inhibiting these molecular mechanisms is significant for preventing occurrence of metastasis.

Cel is a bioactive compound derived from *Tripterygium wilfordii* with a molecular structure of pentacyclic triterpene (Xue et al., 2025). Currently, Cel has exhibited various pharmacological activities, such as anti-obesity, anti-inflammation, and especially, anti-tumor effects (Zhu et al., 2021; Park and Heo, 2024; Zhang et al., 2024). The significant anti-tumor effects of Cel have been reported in diverse preclinical studies, including breast cancer, liver cancer and melanoma (Shi et al., 2025; Kong et al., 2025). The anti-cancer effects of Cel are associated with complex and various mechanisms, which includes enhancing cell apoptosis, suppressing proliferation and migration, and promoting anti-tumor immune response (Zhu et al., 2024). However, whether Cel can regulate the malignant biological characteristics of TNBC cells with anoikis resistance remains unknown.

In this study, we established an anoikis resistant TNBC (TNBC-AR) cell model and found TNBC-AR cells exhibited stronger invasive and migratory ability than normal TNBC cells. Then we investigated the inhibitory effects of Cel on TNBC cells, including significant inhibition on proliferation, colony formation, invasion, migration, aggregation, and anoikis resistance. Mechanistically, we discovered that Cel could suppress the p-EMT state of TNBC-AR cells via reducing the activation of EGFR/MEK/ERK signaling pathway, achieving anti-metastasis effects.

## Materials and methods

### Materials

Cel (purity>98%) was obtained from Shanghai Yuanye Bio-Technology Co., Ltd. (Shanghai, China). Cell counting kit-8 (CCK-8), Matrigel, paraformaldehyde (4%) and dimethyl sulfoxide (DMSO) were derived from LABLEAD Biotechnology Co., Ltd. (Beijing, China). Crystal violet was obtained from Beyotime Biotechnology (Shanghai, China). Annexin V-FITC/PI apoptosis detection kit was supplied by BD Biosciences (San Jose, CA, USA). Primary antibodies to p-EMT markers, EGFR, MEK, p-MEK, ERK, p-ERK were obtained from Proteintech Group, Inc. (Wuhan, China). Antibody to GAPDH was purchased from ABclonal Biotech Co., Ltd. (Wuhan, China). Antibody to p-EGFR was supplied by Abcam (Cambridge, UK).

### Cell culture

Human TNBC cell lines MDA-MB-231 and BT-549 were obtained from Fenghui Biotechnology Co., Ltd. (Hunan, China) and Shanghai Fuheng Biotechnology Co., Ltd. (Shanghai, China) respectively. The MDA-MB-231 and BT-549 cells were incubated in complete Dulbecco’s modified Eagle’s medium (DMEM, Shanghai XP Biomed Ltd., China) and RMPI-1640 (Gibco, USA) supplemented with 1% penicillin-streptomycin (Gibco, USA) and 10% Fetal bovine serum (FBS, Corning, USA) respectively in an incubator with 5% CO_2_ at 37 °C.

### The generation of the anoikis-resistant cell lines

To construct TNBC-AR model, TNBC cells (5 × 10^5^) were seeded in Ultra-Low Attachment 6-well plates (Beyotime Biotechnology, Shanghai, China) for 48 h. The cells transferred to normal plates and re-adherent were considered as TNBC-AR cells. For model construction, cells at passages 3-4 were used. In order to obtain sufficient cell numbers for subsequent experiments, these cells were further expanded in culture. Therefore, the actual experiments were performed using cells at passages 6–8. Before modeling, no significant difference in proliferation rate was observed between the cloned cells and the parental cells; however, the clonogenic capacity declined after prolonged culture (notably beyond passage 11). To confirm the stability of the anoikis-resistant phenotype across passages, we have supplemented the anoikis assay data in Supplementary Figure S1, comparing cells at passage 4 (used for modeling) with those at passages 6 and 8 (used in experiments). The results indicate no significant difference in anoikis rates among passages 4, 6, and 8, supporting the phenotypic stability during the experimental window.

### Cell viability

TNBC cells were seeded into 96-well plates (5000/well) and treated with various concentrations of Cel (0, 0.5, 1.0, 2.0, 4.0, 6.0, 8.0 μM). After culturing for 24h, 10 μL CCK-8 working solution was added. Then the 96-well plates were placed in an enzyme-labeled instrument to measure the absorbance of each well at 450 nm after incubating for 3 h. The absorbance data was recorded and calculated using GraphPad Prism 9.0.

### Cell anoikis assay

Anoikis of TNBC cells was detected by using the Annexin V-FITC/PI apoptosis detection kit. Briefly, cells (3 × 10^5^) were seeded to Ultra-Low Attachment 6-well plates and treated with various concentrations of Cel or EGF for 24 h. Then cells were collected and stained with Annexin V-FITC and PI solution at 25 °C under dark conditions. The anoikis rate of cells was detected using flow cytometer (BD Biosciences, CA, USA).

### Wound healing assay

TNBC cells (5 × 10^5^) were transferred into 6-well plates and then scratched using a 200 μL pipette once they achieved 80% confluence. Subsequently, FBS-free medium with different drugs was used to replace the primary medium. Fluorescent inverted microscope (magnification, ×100) was used to image the wound gap of 0, 24, or 48 h.

### Transwell invasion assay

Invasion assay was detected using 24-well plates and 8.0 μm pore polycarbonate membrane inserts (Corning, USA) coated with 50 μL Matrigel. TNBC cells (5 × 10^4^) were plated on the upper chambers with FBS-free medium containing different concentrations of Cel or EGF. And the lower chambers were supplied with complete medium. After 24h, the TNBC cells that invaded into lower chambers were fixed using 4% paraformaldehyde and stained using crystal violet and eventually imaged and counted in 3 randomly regions using microscope (magnification, ×200).

### Observation of cell morphology

TNBC cells (5 × 10^5^) were seeded in 6-well plates and treated with Cel for 24 h. Cell morphology was imaged using microscope.

### Observation of cell aggregation

TNBC cells (5 × 10^3^) were seeded in Ultra-Low Attachment 96-well plates (Beyotime Biotechnology, Shanghai, China) and treated with Cel for 48 h. Cell aggregation was imaged using microscope.

### Colony formation assay

Colony formation assay was performed as previously described (Wang et al., 2023).

### Bioinformatics analysis

Differential genes regulated by Cel were obtained from SRA database (https://www.ncbi.nlm.nih.gov/sra/, PRJNA1426779). Target genes of TNBC metastasis were obtained from GeneCards database (https://www.genecards.org). EGFR expression profiles were downloaded from GENT2 database (http://gent2.appex.kr/gent2/). The volcano plot, Venn diagram, GO and KEGG analysis, Kaplan-Meier analysis, and EGFR expression of various BC subtypes were plotted using https://www.bioinformatics.com.cn (last accessed on 1 February 2025), an online platform for data analysis and visualization.

### Western blotting

Western blotting was performed as previously described (Li et al., 2025).

### Statistical analysis

GraphPad Prism 9.0 program was used for the statistical analysis. Statistical significances were carried out by unpaired t-tests or one-way ANOVA. P-values ≤0.05 were considered significant. (*P < 0.05, **P < 0.01, ***P < 0.001, ****P < 0.0001). All data were shown as mean ± standard deviation.

## Results

### TNBC cells with anoikis resistance exhibit increased ability to metastasize

**FIGURE 1:**
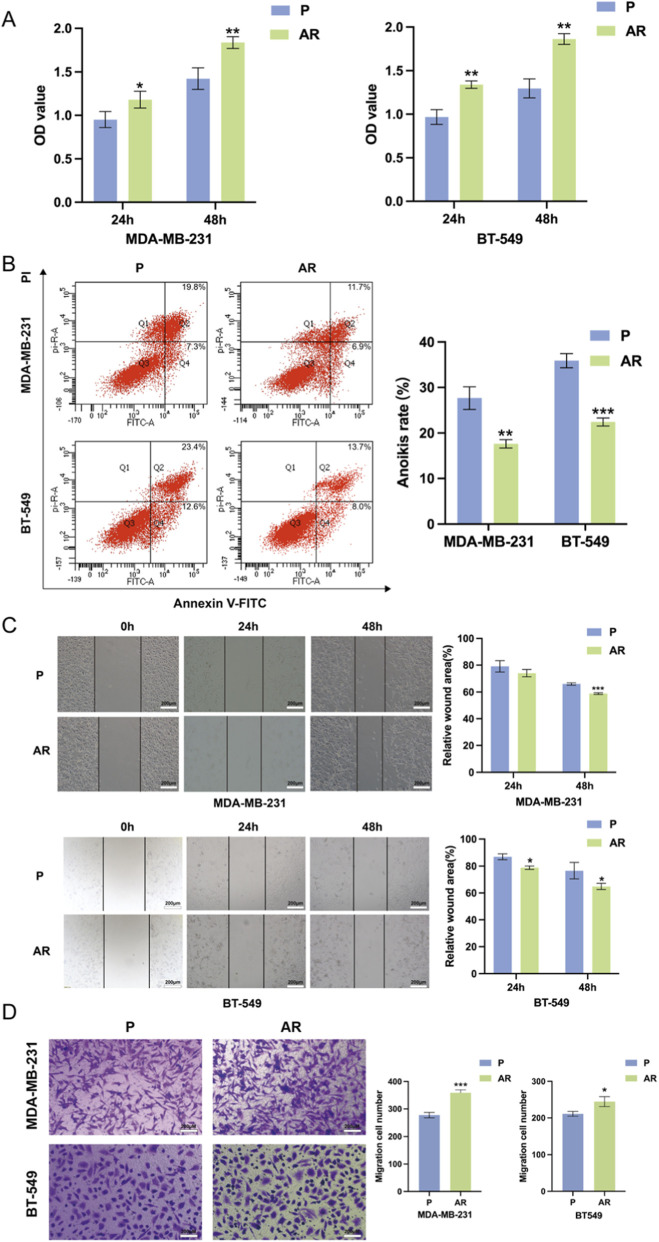
TNBC-AR cells exhibit stronger migration and invasion ability. **(A)** Cell viability in anoikis resistant (AR) and parental (P) MDA-MB-231 and BT-549 cells were detected by CCK-8 after suspension culture for 48 h. **(B)** The anoikis rate of AR and P MDA-MB-231 and BT-549 cells in suspension conditions for 24 h was detected by flow cytometry assay. **(C)** Migration of AR and P MDA-MB-231 and BT-549 cells was determined by wound-healing assay. **(D)** Invasion AR and P MDA-MB-231 and BT-549 cells was determined by Transwell assay.

An anoikis resistant cell model was established using the MDA-MB-231 and BT-549 cell lines as described previously. To investigate the effect of modeling, we used CCK-8 and flowcytometry to access the survival rates and anoikis rates of TNBC-AR cell lines. As shown in Figures 1A,B, the modeling TNBC-AR cells exhibited higher survival rates and lower anoikis rates in suspension conditions, indicating TNBC-AR cells are more resistant to anoikis. Then wound-healing and Transwell assays were conducted to observe the migration and invasion ability of TNBC-AR cells. As shown in Figures 1C,D, the MDA-MB-231-AR and BT-549-AR cells exhibited profound migration and invasion ability compared to the parental (P) cells. These observations indicated that the TNBC-AR cells which survive from ECM detachment are more easily to migrate and invade into distant tissues.

### Cel inhibits the proliferation of TNBC-AR cells

**FIGURE 2:**
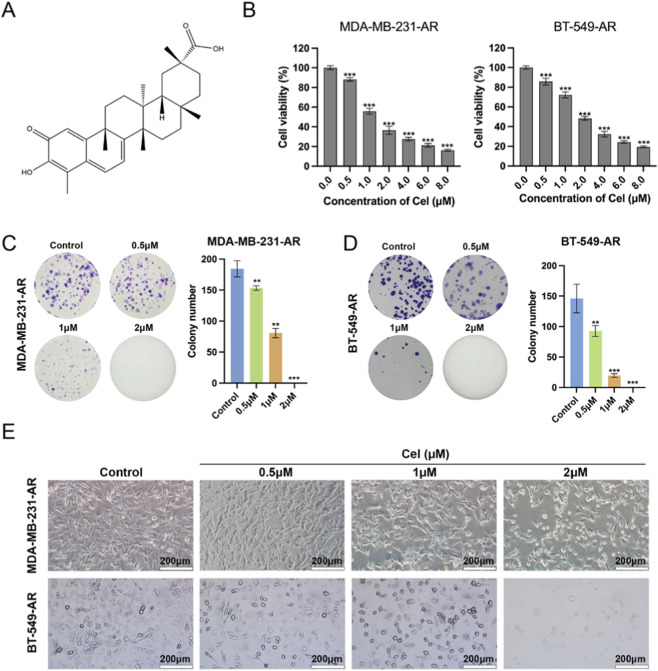
Cel inhibits the proliferation of TNBC cells. **(A)** Chemical structure of Cel. **(B)** Proliferation of MDA-MB-231-AR and BT-549-AR cells was assayed by CCK-8. **(C,D)** Colony-forming ability of MDA-MB-231-AR and BT-549-AR cells was evaluated by colon formation assay. **(E)** Morphological changes of MDA-MB-231-AR and BT-549-AR cells after treatment with Cel for 24 h.

Cel, one of the key biologically active compounds in *Tripterygium wilfordii Hook.* F, shows a pentacyclic triterpene structure as in Figure 2A. A CCK-8 assay was performed to reveal the ability of Cel on suppressing the proliferation of MDA-MB-231-AR and BT-549-AR cells, and Cel at 1 and 2 μM inhibited TNBC-AR cells' viability by nearly 50% (Figure 2B). After calculation, it was obtained that the IC_50_ values of MDA-MB-231-AR and BT-549-AR cells were 1.510 μM and 1.673 μM respectively. Subsequently, we selected concentrations (0.5, 1 and 2 μM) of Cel for further investigation. As shown in Figures 2C,D, Cel inhibited the ability of MDA-MB-231-AR and BT-549-AR cells to form colonies. Besides, treatment with Cel led to lower cell density and more apoptotic morphology changes in MDA-MB-231-AR and BT-549-AR cells (Figure 2E). These results reveal that Cel significantly suppresses the proliferation activity of TNBC-AR cells.

### Cel inhibits the metastasis and enhances the anoikis of TNBC-AR cells

**FIGURE 3:**
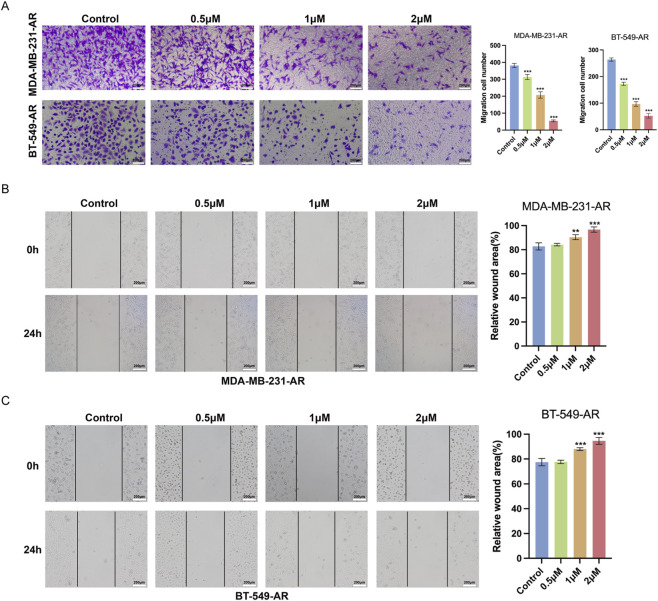
Cel inhibits the invasion and migration of TMBC-AR cells. **(A)** The invasion ability of MDA-MB-231-AR and BT-549-AR cells detected by Transwell assay. **(B,C)** Migration of MDA-MB-231-AR and BT-549-AR cells detected by wound-healing assay.

To investigate the potential impact of Cel on the invasivity and migratory activity of MDA-MB-231-AR and BT-549-AR cells, Transwell assay and wound-healing assay were conducted. As shown in Figure 3A, Cel can suppress the invasive ability of MDA-MB-231-AR and BT-549-AR cells in a dose-dependent manner. Similarly, treatment of Cel dose dependently leads to the suppression of migratory activity of MDA-MB-231-AR and BT-549-AR cells (Figures 3B,C). These results suggest that Cel could inhibit the high metastatic potential of TNBC-AR cells.

### Cel inhibits the aggregation and enhances the anoikis of TNBC-AR cells

**FIGURE 4:**
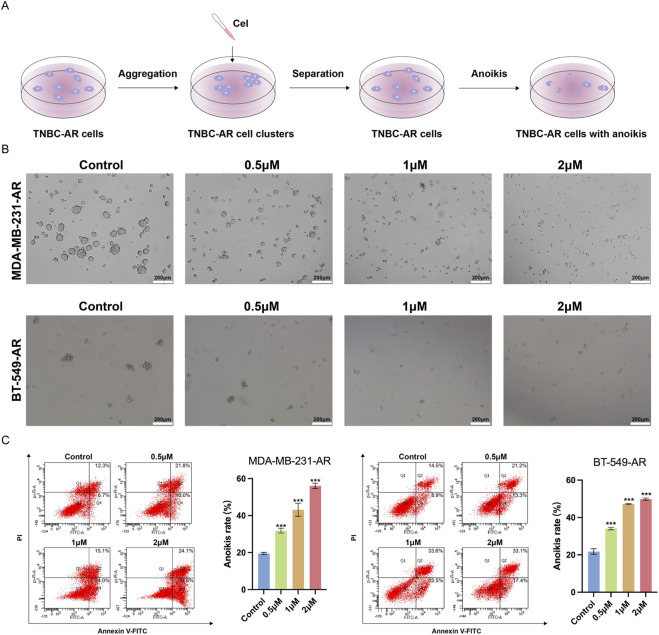
Cel inhibits the aggregation and enhance the anoikis of TNBC-AR cells. **(A)** Schematic illustration of aggregation and anoikis of TNBC-AR cells. **(B)** Cluster formation of MDA-MB-231-AR and BT-549-AR cells. **(C)** The anoikis rate of MDA-MB-231-AR and BT-549-AR cells in suspension conditions for 24 h detected by flow cytometry assay.

Collective migration is a hallmark of cancer metastasis, especially in haematogenous routes (Alexandrova et al., 2025). Compared to single CTC, formation of CTC clusters can provide survival advantage for tumor cells within vascular system (Ildiz et al., 2023). Then we further examined the effect of Cel on the aggregation and cluster formation of TNBC-AR cells in suspension conditions (Figure 4A). As shown in Figure 4B, Cel significantly inhibited the aggregation and formation of CTC clusters in MDA-MB-231-AR and BT-549-AR cells. In addition, Cel enhanced the anoikis of MDA-MB-231-AR and BT-549-AR cells (Figure 4C). And in the treatment of low dose, MDA-MB-231-AR cells were more resistant to anoikis than BT-549-AR cells. These results suggest that Cel could inhibit aggregation and cluster formation of TNBC-AR cells and promote the apoptosis of TNBC-AR cells during the process of metastasis.

### RNA-seq analysis reveals the target of cel

**FIGURE 5:**
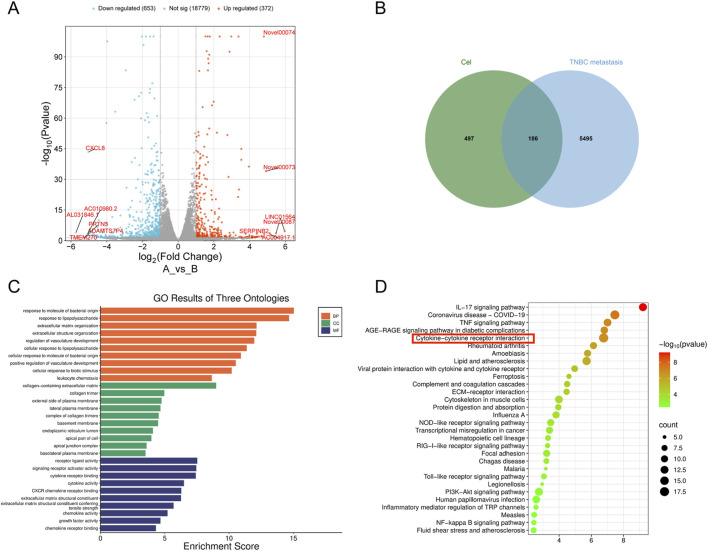
RNA-seq analysis of Cel targeting TNBC metastasis. **(A)** The volcano plot showing the gene expression changes after Cel treatments. **(B)** Venn diagram of common targets between Cel and TNBC with metastasis. **(C)** Top 10 GO enrichment analysis of their ontologies. **(D)** Top 10 potential pathways of KEGG enrichment analysis.

After proving the effects of Cel on TNBC-AR cells metastasis, the signaling pathways and molecular mechanism underlying these effects were explored by transcriptome analysis. The DEG analysis revealed that 419 genes were significantly downregulated, and 234 genes were significantly upregulated after Cel treatment in TNBC cells (Figure 5A). As shown in Figure 5B, 186 common targets between Cel-associated targets and TNBC metastasis-related targets were identified via using GeneCards database. Subsequently, we performed GO and KEGG enrichment analysis of the common targets and the top 10 enriched results are illustrated in Figures 5C,D. Notably, cytokine-cytokine receptor interaction emerged prominently in the KEGG analysis, which includes the EGFR signaling pathway, a signaling pathway that play a crucial role in the anoikis resistance of tumor cells and in the metastasis of TNBC. These results suggest that EGFR signaling pathway may be a significant pathway suppressed by Cel in the process of TNBC metastases.

### Cel may potentially inhibit metastatic traits by interfering with the EGFR/MEK/ERK pathway

**FIGURE 6:**
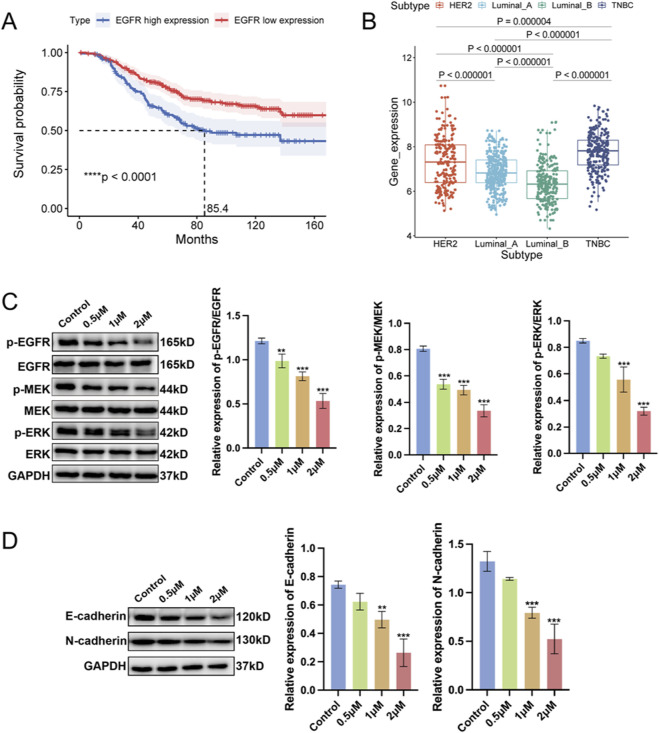
Cel inhibits the activation of EGFR signaling pathway. **(A)** Kaplan-Meier analysis to depict the overall survival of BC patients with different expression of EGFR. **(B)** EGFR expression of various BC subtypes in the GENT2 database. **(C)** Protein expression of EGFR, MEK1/2, ERK1/2 and their phosphorylation detected by Western blot assay. **(D)** Protein expression of p-EMT biomarkers detected by Western blot assay.

To study the underlying clinic relevance of our analysis, we investigated the association of high EGFR expression and BC-specific survival probability, and the expression of EGFR in various subtypes of BC. As shown in Figures 6A,B, BC patients with EGFR high expression exhibits an obvious lower survival probability, and the TNBC subtype exhibits the highest expression of EGFR, which may explain the high mortality of TNBC patients. Subsequently, the effect of Cel on the EGFR signaling pathway was validated in MDA-MB-231-AR cells. As shown in Figure 6C, Cel apparently inhibited the phosphorylation of EGFR (p-EGFR) on the protein level. Furthermore, the downstream molecules of EGFR including p-MEK and p-ERK were significantly reduced after Cel treatment. Besides, E-cadherin and N-cadherin, two biomarkers of p-EMT were both inhibited upon Cel treatment, indicating the suppression of p-EMT state of MDA-MB-231-AR cells (Figure 6D). Collectively, these results reveal that Cel may potentially inhibit metastatic traits by interfering with the EGFR/MEK/ERK pathway and reduce the expression of E-cadherin and N-cadherin.

### Cel inhibits TNBC-AR cells migration and induces the anoikis of TNBC-AR cells via downregulating EGFR/MEK/ERK signaling pathway

**FIGURE 7:**
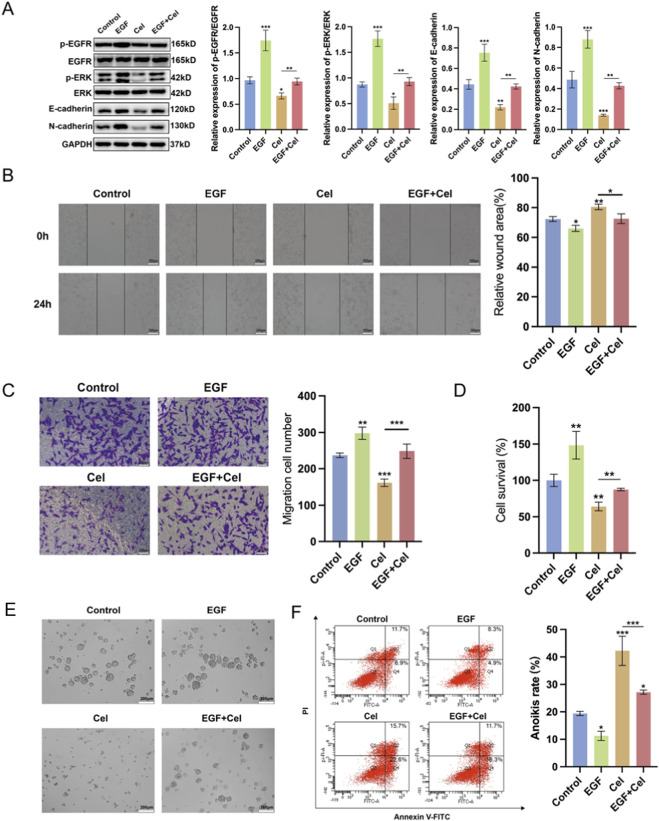
Cel inhibits TNBC-AR cells migration and induces the anoikis of TNBC-AR cells via downregulating EGFR signaling pathway. **(A–F)** MDA-MB-231-AR cells were incubated with Cel (1 μM), EGF (1 ng/mL), or combined treatment of both. **(A)** Western blot assay was performed to detect the protein expression of EGFR, p-EGFR, ERK1/2, p-ERK1/2, E-cadherin and N-cadherin in MDA-MB-231-AR cells. **(B)** Wound healing assay was performed to evaluate the migration of MDA-MB-231-AR cells. The scale bar represents 200 μm. **(C)** Transwell assay was performed to evaluate the invasion of MDA-MB-231-AR cells. The scale bar represents 200 μm. **(D)** CCK-8 assay was performed to evaluate the cell viability of MDA-MB-231-AR cells. **(E)** The cluster formation of MDA-MB-231-AR cells in suspension conditions for 24 h. **(F)** The anoikis rate MDA-MB-231-AR cells in suspension conditions for 24 h was detected by flow cytometry assay.

To further validate the role of EGFR/MEK/ERK signaling pathway in the anti-metastasis effects of Cel treatment, we employed EGFR specific activator EGF protein combined with Cel to treat MDA-MB-231-AR cells in the following study. As shown in Figure 7A, protein levels of EGF and p-EGFR were increased after EGF treatment, and the inhibitory effects of Cel were reversed by EGF, so as the downstream proteins of EGFR. In addition, the Cel-inhibited expression of E-cadherin and N-cadherin was also reversed by EGF, indicating that Cel could impact the p-EMT state via EGFR/MEK/ERK signaling pathway. Moreover, EGF treatment can diminish inhibited invasion and migration ability of MDA-MB-231-AR cells with Cel treatment (Figures 7B,C). Then the effects of EGF on cluster formation and anoikis rate of MDA-MB-231-AR cells were evaluated. As shown in Figures 7D–F, the combined treatment of EGF and Cel reversed the suppressed cluster formation and enhanced anoikis rate compared with only Cel treated group. Taken together, these results indicate that the EGFR/MEK/ERK signaling pathway is profoundly involved in the anti-metastasis effects of Cel.

## Discussion

Breast cancer, the leading cause of cancer associated mortality among women, has become the main form of female tumors with an increasing incidence rate annually (Haque et al., 2024; Yi et al., 2021). As the deadliest subtype of breast cancer, TNBC exhibits distinct malignant features including enormous aggressiveness, high probability to metastasize and high recurrence rate (Wang et al., 2022). Approximately 45% TNBC patients emerge distant metastases to lung or other organs, with the average survival rate less than 18 months (Schmid et al., 2018). However, the lack of approved targeted drug therapy for TNBC metastases contributes to limitation of TNBC treatment (Huang et al., 2019). To improve the symptoms and survival rates of TNBC, potential effective therapy needs to be developed. Here, we investigated the anti-metastasis effect and potential anti-metastasis mechanism of Cel, a naturally occurred anticancer agent isolated from *Thunder God Vine. Celastrol.*

One of the key factors of metastasis is circulating tumor cells (CTCs), the tumor cells that shed from primary tumor site and migrate into circulatory system. To achieve successful distant metastases, CTCs need to survive from anoikis in circulation, a special type of cell apoptosis induced by ECM-detachment, during which CTC clusters were assembled for protective purpose (Han et al., 2021; Kim et al., 2012; Tang et al., 2024). In this study, we established anoikis resistant (AR) TNBC cell lines including MDA-MB-231-AR and BT-549-AR cells, which exhibited more aggressive signatures than parent cells. Our results clearly demonstrated that Cel could notably inhibit the invasion and migration of TNBC-AR cells. It has been observed that CTC clusters formation, which correlate with worse patient prognosis of various tumors, are more resistant to anoikis compared to a single CTC (Han et al., 2021; Maeshiro et al., 2021). Our data suggest that Cel could suppress the formation of CTC clusters in TNBC-AR cells and lead to the increased anoikis rate of TNBC-AR cells.

The EGFR signaling pathway, a crucial regulator of cell survival overexpressed in TNBC, has been found to be tightly associated with metastasis and anoikis resistance of tumor cells (Cicek et al., 2022; Zujun et al., 2023; Guha et al., 2019). In our study, Cel treatment exerted anti-metastatic effects by inhibiting the EGFR/MEK/ERK pathway, thereby reducing the metastatic potential and enhanced anoikis resistance that EGF induces in TNBC-AR cells. In addition, we found that Cel could inhibit the partial-epithelial-to-mesenchymal transition (p-EMT) state of TNBC-AR cells via EGFR/MEK/ERK pathway. P-EMT is a medium state of epithelial-mesenchymal plasticity (EMP), a reversible process from epithelial phenotype to partially or fully mesenchymal phenotype of tumor cells during tumor metastasis, which also includes EMT and mesenchymal-epithelial transition (MET). The tumor cells in p-EMT state exhibit epithelial features and mesenchymal features simultaneously. In our investigation, the expression of epithelial biomarker E-cadherin and mesenchymal biomarker N-cadherin were both observed in TNBC-AR cells, indicating a p-EMT state of TNBC-AR cells. E-cadherin, as a crucial junctional protein mediating cell-to-cell adhesion, is essential for the formation survival of CTC clusters (Balamurugan et al., 2023). This corroborates our observation that the reduction of E-cadherin induced by Cel in TNBC-AR cells was accompanied by suppressed anoikis resistance and inhibition in CTC clusters formation. In addition, tumor cells in mesenchymal phenotype are known to get a reorganized cytoskeleton and to be more motile for stronger ability for invasion and migration (Kyriakopoulou et al., 2022). The expression of N-cadherin explains the high metastatic potential of TNBC-AR cells, which was also inhibited by Cel treatment. Therefore, we deemed that Cel may affect CTC clusters with p-EMT status in TNBC-AR cells by regulating intercellular adhesion and cytoskeleton reorganization, thereby mediating anoikis resistance and high metastatic potential. Although our study provides robust insights through comprehensive *in vitro* analyses, validation in animal models represents an important next step. Due to the inherent complexity of modeling human tumor metastasis in mice, including species-specific differences in tumor-host interactions and the formation of circulating tumor cell clusters with other cells, we are currently developing more physiologically relevant models, such as humanized mouse systems and organoid-based approaches, to validate these findings *in vivo*. Additionally, clinical correlation studies examining relevant clinical samples would help establish the translational relevance of p-EMT and CTCs in human cancer progression.

## Conclusion

In conclusion, we used an anoikis resistance model to explore the anti-metastatic ability and mechanisms of Cel. Our results suggest that Celastrol may suppress anoikis resistance and the metastatic potential of TNBC-AR cells, at least in part, by disrupting CTC cluster formation. Mechanistically, the observed effects were accompanied by reduced activity of the EGFR/MEK/ERK pathway and could be partially reversed by EGF. This implicates the involvement of EGFR signaling in the anti-metastatic activity of Cel. Besides, we verified that Cel suppressed the p-EMT state of TNBC-AR cells, which are crucial for maintaining the metastatic potential of tumor cells in circulation. This research provides potential insights for the development of metastatic TNBC treatments and may offer new research ideas for exploring the molecular mechanisms of tumor metastases.

## Data Availability

The data presented in the study are deposited in the NCBI (SRA) repository, accession number PRJNA1426779.
