# Supplementary material for: Celastrol increases anoikis sensitivity to suppress triple-negative breast cancer via EGFR pathway and p-EMT state regulation
Source: Front Pharmacol. 2026 Mar 2;17:1747871. doi: 10.3389/fphar.2026.1747871 (PMC12989539; doi:10.3389/fphar.2026.1747871)
Supplement: Supplementary file 1 [file Image1.pdf]

Supplementary Materials

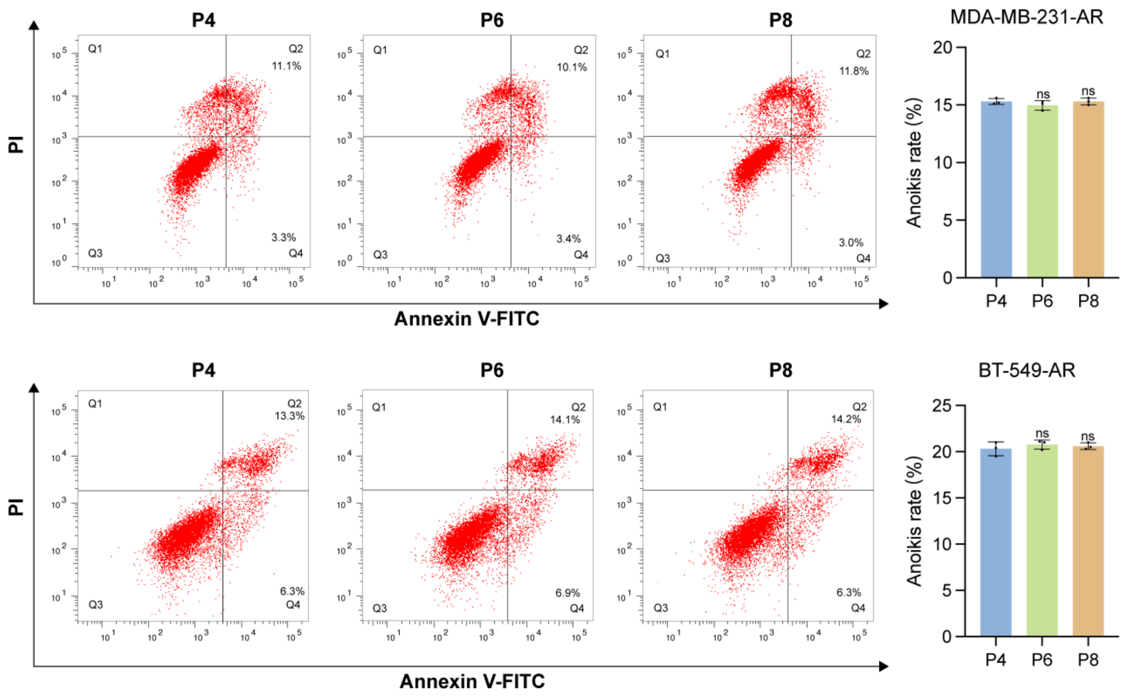

Fig. S1. The stability of the AR phenotype over passages. To confirm the stability of the anoikis-resistant phenotype across passages, we have compared cells at passage 4 (obtained after modeling) with those at passages 6 and 8 (used in experiments). The results indicate no significant difference in anoikis rates among passages 4, 6, and 8, supporting the phenotypic stability during the experimental window.
